# The Association Between Serum Uric Acid Levels and the 10‐Year Prospective Risk of Dyslipidemia: A Cohort Study From Healthy Heart Project (YHHP)

**DOI:** 10.1002/hsr2.70857

**Published:** 2025-05-22

**Authors:** Hamidreza Mohammadi, Seyed Reza Mirjalili, Pedro Manuel Marques‐Vidal, Marzieh Azimizadeh, Seyede Mahdiah Nemayandah, Mohammadtaghi Sarebanhassanabadi, Sedighe Sadeghi

**Affiliations:** ^1^ Yazd Cardiovascular Research Center, Non‐communicable Diseases Research Institute Shahid Sadoughi University of Medical Sciences Yazd Iran; ^2^ Department of Medicine, Internal Medicine Lausanne University Hospital and University of Lausanne Lausanne Switzerland; ^3^ Department of Epidemiology Shahid Sadoughi University of Medical Sciences Yazd Iran

**Keywords:** cohort study, dyslipidemia, serum uric acid

## Abstract

**Background and Aims:**

Dyslipidemia is a major risk factor for cardiovascular disease (CVD) and is increasingly prevalent globally, particularly in developing countries undergoing lifestyle transitions. Serum uric acid (SUA) has been implicated in various metabolic disorders, including dyslipidemia. This study aimed to evaluate the association between high SUA and the 10‐year prospective risk of dyslipidemia.

**Methods:**

This cohort study uses data from the Yazd Healthy Heart Project (YHHP). Participants were followed for 10 years, during which the association between dyslipidemia incidence and SUA levels was assessed using multivariate Cox proportional hazard models.

**Results:**

Among 693 participants analyzed, higher SUA quartiles were associated with older age, male gender, and adverse anthropometric and metabolic profiles. Elevated SUA levels correlated positively with triglycerides (*r* = 0.21, *p* < 0.001), total (*r* = 0.13, *p* < 0.001) and low‐density lipoprotein cholesterol (*r* = 0.12, *p* = 0.001) levels, but not with high‐density lipoprotein cholesterol (*r* = 0.07, *p* > 0.05). After adjusting for age, lifestyle factors, and metabolic parameters, the incidence of dyslipidemia increased across SUA quartiles in males, with adjusted hazard ratios of 1.33 (95% CI: 0.90–1.97), 1.66 (95% CI: 1.10–2.54), and 1.79 (95% CI: 1.17–2.72) for the second, third, and fourth quartiles of SUA, respectively (*p* for trend 0.001), but not in females: 1.11 (95% CI: 0.59–2.06), 0.95 (95% CI: 0.55–1.64), and 1.14 (95% CI: 0.60–2.16), respectively, *p* for trend 0.86.

**Conclusion:**

Elevated SUA levels independently associated with the development of dyslipidemia over a decade in an Iranian population. The findings underscore the potential utility of SUA as a biomarker for assessing dyslipidemia risk, particularly in men. Further research should explore mechanistic pathways linking SUA to dyslipidemia and evaluate interventions targeting SUA reduction to mitigate dyslipidemia risk.

**Abbreviations**
BMI: body mass index
CVD: cardiovascular disease
DBP: diastolic blood pressure
DM: diabetes mellitus
FBS: fasting blood sugar
HC: hip circumference
HDL‐C: high‐density lipoprotein cholesterol
LDL‐C: low‐density lipoprotein cholesterol
MetS: metabolic syndrome
SBP: systolic blood pressure
SUA: serum uric acid
TC: total cholesterol
TG: triglycerides
WC: waist circumference
WHR: waist/hip ratio
YHHP: Yazd Healthy Heart Project

## 1 Introduction

Dyslipidemia is a preventable risk factor for cardiovascular disease (CVD), which is one of the main causes of death all over the world [1]. Additionally, it is closely associated with obesity, diabetes mellitus (DM), metabolic syndrome (MetS), and nonalcoholic fatty liver disease. These conditions provide substantial public health issues on a global basis [2, 3]. The occurrence of dyslipidemia has increased in numerous developing countries in recent years as a result of lifestyle modifications linked to economic progress [4]. The substantial increase in overweight and obesity rates, particularly among adolescents, is likely to lead to a higher incidence of DM and dyslipidemia in the near future. Iran, a country with a population of over 80 million, primarily concentrated in urban areas, has a significant issue of poor metabolic health. Hence, it is imperative to manage dyslipidemia to decrease the incidence of premature deaths caused by CVDs in the country and achieve a third reduction in mortality by 2030 [5].

Serum uric acid (SUA) is the final product of purine metabolism, originating from both endogenous and exogenous sources [6]. Endogenous purines, which constitute 80% of total body purines, result mainly from the breakdown of nucleic acids within the body [7]. Exogenous purines are derived from dietary sources like seafood, fatty meats, organ meats (e.g., liver, kidney), fructose, and alcohol [8]. Factors such as dietary habits, lifestyle choices, and medication use can influence SUA levels [9, 10]. Both genetic predisposition and environmental factors contribute to the variability in SUA levels [11]. Over recent decades, there has been a rising prevalence of hyperuricemia observed in Western countries as well as in China [12, 13].

Epidemiological studies have demonstrated that elevated SUA levels are increasingly associated with hypertension, CVD, and MetS [14, 15]. An elevated SUA level is associated with increased risks of both all‐cause mortality and cardiovascular mortality [16, 17]. The associations between SUA and risk factors for CVD make it difficult to determine if SUA directly causes these problems or just acts as an indicator for persons who are at higher risk. SUA levels are linked to other conventional risk factors such as blood lipids, MetS, and DM [18]. The exact function of SUA in these conditions is still a matter of disagreement and is now being discussed, as it can often be linked with other risk factors such as diet, dyslipidemia, and obesity. Increasing evidence indicates that hyperuricemia, or high levels of SUA, is related to MetS and its component parts, even when SUA levels are within the normal range. The correlation between SUA and dyslipidemia is complicated and yet not entirely explained [11]. There are limited studies investigating the relationship between SUA levels and lipid profiles in adult populations from India [18], Italy [19], and the USA [11]. However, recent studies suggesting a link between SUA levels and dyslipidemia remain controversial. Therefore, this study aimed to investigate the association between SUA levels and dyslipidemia in a large‐scale Iranian population participating in the Yazd Healthy Heart Project cohort.

## 2 Materials and Methods

### 2.1 Study Design and Study Subjects

This cohort study aimed to determine if high SUA levels contribute to the development of dyslipidemia. The research used data from the YHHP, involving 2000 individuals aged 20–74 years. These participants were part of the YHHP, an epidemiological study focused on CVDs and metabolic disorders within the general population. The participants were selected from the urban population of Yazd city, the capital of Yazd province, located in central Iran. Detailed information about the YHHP has been previously published, along with a previous study based on this cohort database [20, 21].

### 2.2 Included Participants

At baseline, the prevalence of dyslipidemia was 47.2% (*n* = 944). As a result, all participants who had not been diagnosed with dyslipidemia at baseline (2005–2006) (*n* = 1056) were included in the current study. These individuals were followed for 10 years and reassessed for dyslipidemia during 2015–2016. Of the 1056 participants who underwent the baseline examination, 78 were excluded due to death during the study, and 285 were excluded due to missing data. The remaining 693 participants were included in the present study.

### 2.3 Data Collection

The questionnaire included demographic characteristics, education, physical activity, and smoking, which were obtained with face‐to‐face interviews. Participants were categorized based on their educational attainment into three groups: primary, high school, and academic education. Physical activity levels were assessed using the International Physical Activity Questionnaire (IPAQ), which classified participants into low, moderate, and high activity groups.

Based on their current smoking status, the participants were divided into two categories: smokers and non‐smokers. Blood pressure was measured following the YHHP cohort protocol using an automatic digital blood pressure monitor (Omron, M6 Comfort, Osaka, Japan). Weight (kg), height (cm), waist circumference (WC), hip circumference (HC), waist/hip ratio (WHR), Body mass index (BMI), as anthropometric markers were measured based on the cohort study protocol. Dyslipidemia was defined as TC ≥ 240 mg/dL, LDL‐C ≥ 160 mg/dL, HDL‐C < 40 mg/dL, and TG ≥ 200 mg/dL [22] or medical treatment or confirmed dyslipidemia by a physician.

### 2.4 Biochemical Measurements

Blood samples were collected from the antecubital vein after a 12‐h fasting period. Following centrifugation, serum was isolated for biochemical analysis. SUA and fasting blood sugar (FBS) were measured using Pars Azmoon kits (Pars Azmoon Inc., Tehran, Iran), while the lipid profile, including triglycerides (TG), total cholesterol (TC), high‐density lipoprotein cholesterol (HDL‐C), and low‐density lipoprotein cholesterol (LDL‐C), was measured using Bionic kits (Bionic Company, Tehran, Iran). The analyses were conducted using a biochemical auto analyzer (BT 3000, Italy) at the medical laboratory of Afshar Hospital in Yazd, Iran.

### 2.5 Ethical Consideration

The study was performed in accordance with the guidelines of the Declaration of Helsinki. The Shahid Sadoughi University of Medical Sciences ethics committee approved the present study (Ethic code IR.SSU.REC.1402.105). Eligible participants were included in the study after signing the informed consent form. The data collection process ensured complete anonymity, with no personally identifiable information included in the individual data. This study adhered to the Strengthening the Reporting of Observational Studies in Epidemiology (STROBE) guidelines [23].

### 2.6 Statistical Analysis

The statistical analysis was conducted using IBM SPSS version 23 (SPSS Inc., Chicago, IL, United States). Continuous variables were reported as the mean and standard deviation. The categorical variables were shown as frequency and percentage. The correlation between lipid profiles and SUA levels was evaluated using Pearson's correlation test. A one‐way analysis of variance (ANOVA) was conducted to find out the differences among the groups. A quantile‐based analysis was conducted by dividing the levels of SUA into quartiles, with the lowest quartile being established as the reference. The threshold levels of SUA quartiles were determined based on gender as follows. For males: Quartile 1 (Q1) < 3.82 mg/dL, 3.82 ≤ Q2 < 4.6 mg/dL, 4.6 ≤ Q3 < 5.37 mg/dL, and 5.37 mg/dL ≤ Q4. For females: Quartile 1 (Q1) < 2.95 mg/dL, 2.95 ≤ Q2 < 3.5 mg/dL, 3.5 ≤ Q3 < 4.1 mg/dL, and 4.1 mg/dL ≤ Q4. Multivariable Cox proportional hazard models were employed to estimate the risk of new‐onset dyslipidemia. Three models were evaluated: Model I was adjusted for age and sex; Model II included additional adjustments for smoking, physical activity, and education; and Model III was further adjusted for BMI, WHR, FBS, SBP, and DBP. A two‐sided *p*‐value of less than 0.05 was considered as statistically significant.

## 3 Results

### 3.1 Clinical Characteristics of the Participants

A total of 693 participants (mean age 44.57 ± 14.31 years; 54.3% males) were enrolled in this study. The individuals were categorized into quartiles according to their SUA levels, and their characteristics were compared among these quartiles. Participants in the higher SUA quartiles tended to be older, more likely to be male, and had higher weight, BMI, WC, WHR, SBP, DBP, TG, total cholesterol, and LDL‐C levels, but lower HDL‐C levels (Table 1).

**Table 1:** Characteristics of the study population according to quartiles of serum uric acid.

	Serum uric acid quartiles				
	Q1 *N* = 190	Q2 *N* = 158	Q3 *N* = 182	Q4 *N* = 163	*p*‐value
**Age (years)**	41.3 ± 12.9	44.2 ± 13.6	46.2 ± 15.4	46.9 ± 14.7	0.001
**Mean follow‐up (years)**	9.9 ± 1.0	10.1 ± 0.9	9.8 ± 1.0	9.8 ± 0.9	0.027
**Male (%)**	45 (23.7%)	74 (46.8%)	124(68.1%)	133 (81.6%)	0.001
**Education (%)**					0.17
Primary	100 (53.5%)	78 (50.6%)	91 (52.9%)	64 (41%)	
High school	73 (39%)	57 (37%)	61 (35.5%)	71 (45.5%)	
Academic	14 (7.5%)	19 (12.3%)	20 (11.6%)	21 (13.5%)	
**Anthropometry**					
Weight (kg)	65.2 ± 11.6	69.2 ± 11.1	70.8 ± 11.6	77.3 ± 12.3	< 0.001
WHR	0.87 ± 0.10	0.88 ± 0.09	0.90 ± 0.07	0.92 ± 0.08	< 0.001
WC (cm)	88.5 ± 11.8	90.0 ± 11.2	91.3 ± 12.0	96.0 ± 12.7	< 0.001
HC (cm)	100.7 ± 11.1	102.6 ± 9.9	101.2 ± 9.1	103.9 ± 9.2	0.011
Waist	88.5 ± 11.8	89.0 ± 11.2	91.3 ± 12.0	96.0 ± 12.7	< 0.001
BMI (kg/m^2^)	24.95 ± 4.36	25.43 ± 4.32	25.41 ± 4.36	26.68 ± 3.90	0.002
**Current smokers (%)**	18 (9.5%)	30 (19.0%)	32 (17.6%)	33 (20.2%)	0.024
**Physical activity (%)**					0.09
Low	57 (47.1%)	66 (50.4%)	83 (59.7%)	74 (56.5%)	
Moderate	17 (14.0%)	10 (7.6%)	10 (7.2%)	18 (13.7%)	
High	47 (38.8%)	55 (42.0%)	46 (33.1%)	39 (29.8%)	
**Blood pressure (mm Hg)**					
SBP	121 ± 14	124 ± 14	126 ± 14	130 ± 15	0.001
DBP	79 ± 9	80 ± 8	82 ± 7	84 ± 9	0.001
**Blood levels (mg/dL)**					
FBS	97.5 ± 45.98	97 ± 34.2	91.57 ± 23.25	92.38 ± 20.37	0.2
TC	172.4 ± 31.1	178.5 ± 30.4	180.8 ± 33.5	185.0 ± 30.2	0.002
LDL‐C	92.7 ± 28.1	99.4 ± 25.1	97.5 ± 31.2	104.2 ± 29.0	0.002
TG	107.2 ± 42.6	109.0 ± 35.9	122.3 ± 40.9	129.3 ± 39.6	< 0.001
HDL‐C	57.6 ± 11.6	57.3 ± 10.9	58.1 ± 11.6	55.4 ± 10.5	0.12

WHR, waist/hip ratio; WC, waist circumference; HC, hip circumference; BMI, body mass index; SBP, systolic blood pressure; DBP, diastolic blood pressure; FBS, fasting blood sugar; TC, total cholesterol; LDL‐C, low‐density lipoprotein cholesterol; TG, triglycerides; HDL‐C, high‐density lipoprotein cholesterol. Total participants were classified into quartiles according to their SUA levels. Quartile 1 (Q1) < 3.4 mg/dL, 3.4 ≤ Q2 < 4.1 mg/dL, 4.1 ≤ Q3 < 4.9 mg/dL, 4.9 mg/dL ≤ Q4. For Females: Quartile 1 (Q1) < 2.95 mg/dL, 2.95 ≤ Q2 < 3.5 mg/dL, 3.5 ≤ Q3 < 4.1 mg/dL, 4.1 mg/dL ≤ Q4.

### 3.2 Association of SUA With Incidence of Dyslipidemia and Lipid Profiles

**Figure 1:**
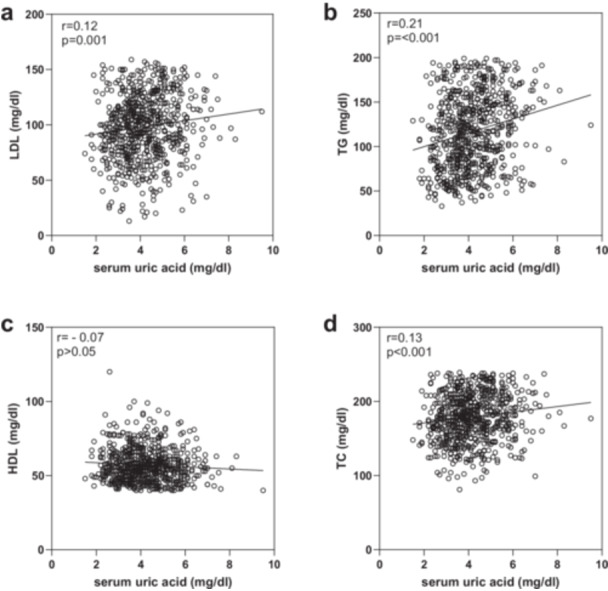
Correlations between serum uric acid and LDL (a), triglycerides (TG, b), HDL (c), and total cholesterol (TC, d). The scales on the Y‐axis vary across the figures.

Among the 693 participants included, 456 matched the diagnostic criteria for dyslipidemia. Out of a total of 456 participants who had dyslipidemia, 135 (29.6%) had hypertriglyceridemia, 110 (24.12%) had hypercholesterolemia, 283 (62%) had low HDL cholesterolemia, and 103 (22.58%) had hyper LDL cholesterolemia. Pearson correlation analysis showed that SUA levels had a positive correlation with TG, TC, and LDL levels (*p* < 0.01), although no significant correlation was found between SUA and HDL levels (Figure 1).

The overall incidence of newly diagnosed dyslipidemia throughout the follow‐up period was 65.8%. The incidence of newly developed dyslipidemia was 57.9%, 64.6%, 68.1%, and 73.6% in quartiles 1–4, respectively. In comparison to the Q1 group, the hazard ratio (HR) and 95% confidence interval (CI) for the occurrence of dyslipidemia in Model I were 1.016 (0.77–1.33), 1.48 (1.13–1.94), and 1.7 (1.27–2.27) in the Q2, Q3, and Q4 groups, respectively. In the gender‐specific analysis, it was found that the incidence of dyslipidemia increased across SUA quartiles in males, with adjusted hazard ratios of 1.33 (95% CI: 0.90–1.97), 1.66 (95% CI: 1.10–2.54), and 1.79 (95% CI: 1.17–2.72) for the second, third and fourth quartiles of SUA, respectively (*p* for trend 0.001), but not in females: 1.11 (95% CI: 0.59–2.06), 0.95 (95% CI: 0.55–1.64), and 1.14 (95% CI: 0.60–2.16), respectively, *p* for trend 0.86 (Table 2).

**Table 2:** Risk of dyslipidemia according to quartiles of serum uric acid, overall and stratified by gender.

	Q1	Q2	Q3	Q4	*p* for trend
**All participants**					
Crude	1	0.99 (0.76–1.30)	1.43 (1.10–1.85)	1.61 (1.24–2.10)	< 0.001
Model I	1	1.02 (0.77–1.33)	1.48 (1.13–1.94)	1.70 (1.27–2.27)	< 0.001
Model II	1	1.16 (0.84–1.60)	1.45 (1.04–2.05)	1.72 (1.20–2.44)	0.002
Model III	1	1.09 (0.78–1.51)	1.52 (1.06–2.18)	1.78 (1.21–‐2.62)	0.001
**Men**					
Crude	1	1.29 (0.91–1.82)	1.51 (1.05–2.16)	1.64 (1.16–2.31)	0.003
Model I	1	1.28 (0.90–1.81)	1.50 (1.05–2.15)	1.65 (1.17–2.34)	0.003
Model II	1	1.27 (0.87–1.86)	1.46 (0.98–2.17)	1.66 (1.12–2.45)	0.009
Model III	1	1.33 (0.90–1.97)	1.66 (1.10–2.54)	1.79 (1.17–2.72)	0.004
**Women**					
Crude	1	0.78 (0.51–1.19)	0.81 (0.54–1.22)	1.42 (0.96–2.12)	0.084
Model I	1	0.79 (0.52–1.21)	0.79 (0.53–1.19)	1.29 (0.84–1.95)	0.25
Model II	1	0.94 (0.53–1.69)	0.94 (0.57–1.58)	1.15 (0.65–2.06)	0.67
Model III	1	1.11 (0.59–2.06)	0.95 (0.55–1.64)	1.14 (0.60–2.16)	0.86

The results are expressed as hazard ratios and 95% confidence intervals. Model I: adjusted for age and sex. Model II: adjusted for variables included in Model I and smoking, physical activity, and education. Model III: adjusted for variables included in Model II and FBS, BMI, WHR, SBP, and DBP. Participants were classified into quartiles according to their SUA levels. For males: Quartile 1 (Q1) < 3.82 mg/dL, 3.82 ≤ Q2 < 4.6 mg/dL, 4.6 ≤ Q3 < 5.37 mg/dL, 5.37 mg/dL ≤ Q4. For Females: Quartile 1 (Q1) < 2.95 mg/dL, 2.95 ≤ Q2 < 3.5 mg/dL, 3.5 ≤ Q3 < 4.1 mg/dL, 4.1 mg/dL ≤ Q4.

## 4 Discussion

Our study was a part of a cohort study with a median of 10 years of follow‐up conducted for evaluating CVDs and metabolic disorders in central Iran. To the best of our knowledge, there are few studies which focused on the incidence of the dyslipidemia at different levels of SUA in a representative sample of healthy individuals. Several important implications can be drawn from our research. First, SUA level was positively correlated with TG, TC, and LDL levels. Second, anthropometric parameters such as weight, BMI, WC, and WHR were higher in subjects with higher SUA concentration. Third, elevated baseline SUA concentration was significantly associated with the increased the risk of dyslipidemia in healthy individuals after adjusting for potential confounders. Fourth, when the analysis was further stratified by gender, the association was observed only in the group of male participants.

Our findings align with several prospective studies indicating that elevated SUA levels increase the risk of dyslipidemia [24, 25, 26]. Consistent with previous cross‐sectional [26, 27, 28] and prospective [29, 30] studies conducted among Chinese, Japanese, Portuguese [31], and American populations [32], our results reinforce this association between elevated SUA and dyslipidemia. For instance, Zheng et al. demonstrated in a longitudinal study that high SUA levels significantly increased the risk of hypertriglyceridemia (hazard ratio, 1.43) over an 8‐year follow‐up period [33]. But these studies did not show the relationship between dyslipidemia and uric acid.

- The relationship between SUA concentration and blood lipid profiles varies across different populations [34, 35, 36]. In our study, we found that SUA levels were associated with TG, TC, LDL‐C, and non‐HDL. Recent research across diverse populations has demonstrated a significant correlation between SUA levels and TG, while the association with HDL remains inconclusive. Previous studies have found a correlation between SUA and TG, consistent with our findings [37, 38]. It is hypothesized that TG synthesis requires NADPH, leading to increased SUA production [11]. Uric acid promotes oxidative stress intracellularly by activating NADPH oxidase, which attaches to the mitochondrial membrane, causing mitochondrial oxidative stress. This stress induces pro‐inflammatory signaling and stimulates the innate immune system, perhaps elucidating how uric acid induces subclinical inflammation in non‐crystal‐forming hyperuricemia [31]. The exact biological mechanism linking SUA to dyslipidemia is not fully understood, but some authors propose that uric acid has pro‐inflammatory effects by stimulating various inflammatory molecules [39]. Additionally, SUA can inhibit adiponectin synthesis in adipocytes by reducing nitric oxide production in arterial endothelial cells, disrupting the tricarboxylic acid cycle and fatty acid β‐oxidation, and promoting cellular oxidative activity [40], which reduce the decomposition of serum triglycerides, leading to the higher incidence of hypertriglyceridemia in participants with high SUA levels.

Several epidemiological studies have indicated that SUA levels were higher in males compared to females [41, 42], with levels increasing post‐menopause [43]. Our study similarly found a higher proportion of males in the higher SUA quartiles, although we did not differentiate between pre‐ and post‐menopausal women. Interestingly, while a relationship between SUA levels and dyslipidemia incidence was observed in men, this association was not significant in women. This suggests that gender may play a crucial role in this association. Most studies have suggested that the link between SUA and dyslipidemia is not evident in females [33]. Although the specific mechanisms underlying the gender‐related role of uric acid in dyslipidemia risk are not fully understood, research by Shi et al. [44] and Techatraisak et al. [45] indicated that the association between SUA levels and dyslipidemia exists in post‐menopausal women but not in premenopausal women. Estrogen in premenopausal women augments uric acid excretion and elevates renal clearance. Therefore, it is likely that endogenous estrogen contributes to the absence of a relationship between SUA levels and dyslipidemia in premenopausal women [46].

Prior research has shown a relationship between SUA levels and body weight, as well as a positive link between SUA levels and BMI in healthy individuals, a finding also observed in this study [47]. Significant positive relationships between SUA levels and obesity have been observed in populations from China [48], Japan [49], India [50], Pakistan [51], Iraq [52], and the United States [53]. In our study, BMI, WC, and WHR were associated with elevated SUA levels; however, the mechanisms by which uric acid levels increase in obesity are not well understood. It is hypothesized that this may involve both overproduction and poor renal excretion of uric acid.

### 4.1 Clinical and Public Health Potential

The results of the YHHP cohort study have shown that a large part of the population of Yazd city in the center of Iran is exposed to dyslipidemia as one component of the metabolic syndrome, followed by the occurrence of cardiovascular events [54]. Along with encouraging to increase physical activity and follow diets, the use of biomarkers can help predict the occurrence of dyslipidemia and prevent the occurrence of these disorders. The potential of using uric acid as a biomarker to predict the progression of dyslipidemia should be further explored, as uric acid has previously been suggested as a pro‐inflammatory molecule that may mediate subclinical inflammation.

### 4.2 Strengths and Limitations

Several strengths and potential limitations of the study deserve comment. The main strengths include a long‐term follow‐up and control for numerous potential confounding variables. The community‐based prospective design minimizes the chances of reverse causation and recall bias. Additionally, our study included a broader age range, enhancing generalizability compared to previous studies that focused solely on middle‐aged and elderly individuals. This study was conducted for the first time in the center of Iran, but the findings may not be fully generalizable to other populations with different ethnicities. In the study of Liu et al., it was found that ethnic diversity has an effect on the relationship between uric acid and dyslipidemia [55]. Besides genetic differences, there are also lifestyle differences between different ethnicities. The study did not differentiate between pre‐ and post‐menopausal women, which could be an important factor given the hormonal changes that affect SUA and lipid levels. Dietary pattern changes were not evaluated during this study, and it is better to evaluate them in future studies.

## 5 Conclusion

The present study shows that, in a healthy population, a high baseline level of SUA is strongly associated with the development of dyslipidemia over a 10‐year period. Prevention and early treatment of hyperuricemia can reduce the incidence of dyslipidemia as one component of metabolic syndrome. SUA has potential utility as a biomarker for predicting future dyslipidemia incidence in initially healthy individuals. However, further studies are suggested to explore the impact of nutritional interventions and dietary modifications on the association between uric acid levels and dyslipidemia.

## Author Contributions

**Hamidreza Mohammadi:** writing – original draft, writing – review and editing, project administration, formal analysis, data curation, methodology, conceptualization. **Seyed Reza Mirjalili:** writing – review and editing, investigation, formal analysis, software. **Pedro Manuel Marques‐Vidal:** writing – review and editing, supervision, investigation, validation. **Marzieh Azimizadeh:** writing – original draft, formal analysis, data curation, methodology, investigation, project administration, writing – review and editing, conceptualization. **Seyede Mahdiah Nemayandah:** validation, methodology, software, formal analysis, supervision, writing – review and editing. **Mohammadtaghi Sarebanhassanabadi:** conceptualization, investigation, writing – review and editing, writing – original draft, project administration. **Sedighe Sadeghi:** writing – review and editing, investigation.

## Ethics Statement

The study was performed in accordance with the guidelines of the Declaration of Helsinki. The Shahid Sadoughi University of Medical Sciences ethics committee approved the present study (Ethic code IR.SSU.REC.1402.105). Eligible individuals were included in the study after completing the informed consent form. The data collection process ensured complete anonymity, with no personally identifying information included in the individual data. This study was based on the Strengthening the Reporting of Observational Studies in Epidemiology (STROBE) declaration.

## Consent

All participants have signed an informed consent which includes the consent for publication.

## Conflicts of Interest

The authors declare no conflicts of interest.

## Transparency Statement

The lead author Mohammadtaghi Sarebanhassanabadi affirms that this manuscript is an honest, accurate, and transparent account of the study being reported; that no important aspects of the study have been omitted; and that any discrepancies from the study as planned (and, if relevant, registered) have been explained.

## Data Availability

The data that support the findings of this study are available from the corresponding author upon reasonable request.
